# “If I had stayed back home, I would not be alive any more…” – Exploring end-of-life preferences in patients with migration background

**DOI:** 10.1371/journal.pone.0175314

**Published:** 2017-04-06

**Authors:** Piret Paal, Johannes Bükki

**Affiliations:** 1 Department of Research and Development, Hospice Care DaSein, Munich, Germany; 2 Institute of Nursing Sciences and Palliative Care, Paracelsus Medical University, Salzburg, Austria; Missouri Botanical Garden, UNITED STATES

## Abstract

**Background:**

In patients with life-limiting conditions and a history of migration, a higher risk of not dying at home and limited access to palliative care services has been reported.

**Aim:**

To explore the views and end-of-life preferences of patients with a migration history in Germany and to identify migration specific themes.

**Design:**

Two-armed study using Kaufmann’s ‘understanding interview’ (‘focused interview’) method and grounded theory approach. Thematic content analysis was applied using MaxQDA 12 software.

**Setting/Participants:**

Migrant and non-migrant adult patients with far advanced, life-limiting disease receiving palliative care in different specialist level settings (specialist home palliative care, palliative care inpatient unit, inpatient hospice).

**Results:**

The 37 interviewees (19 native Germans and 18 patients from Europe and the U.S., Israel, Turkey, and Indonesia) expressed eleven themes covering health care- and patient-related issues, of which four emerged to be specific for migrants: worse survival in home country; the perception of an altered identity and ‘not belonging’; language skills as prerequisite to survive; and longing for ‘home’ while being attached to Germany. From these categories, three overarching themes were derived: (1) a limited understanding of the concept of ‘palliative care’; (2) the suppression of end of life discussions for its association with suffering and loss of autonomy; and (3) the significance of complex individual migration histories.

**Conclusions:**

Based on these findings, the concept of a ‘double home’ experience is proposed. Barriers to access to palliative care should be minimized for all patients while cultural stereotyping has to be avoided.

## Introduction

Research activity on cultural preferences and barriers in palliative care has increased during the last years[1, 2]; however, studies involving patients directly are rare and doing research in this vulnerable group may be challenging. Health care professionals who fear additional burden to the patient, doubt the relevance of research, or have ethical objections practice ‘gatekeeping’[3], while poor health and uncertain prognosis[4] may be barriers on an individual level. Thus, patient-level data are scarce and evidence regarding patients’ cultural needs in palliative care is weak. In a number of qualitative studies, negative interactions have been documented between individual factors such as social status, level of education, or language fluency, and the limited ability of the health care system to address cultural and social issues[5]. A survey among Dutch physicians revealed differences in end-of-life (EoL) care and decision making between Dutch natives and non-western migrants[6], and a population based UK study reported variation regarding the place of cancer deaths between ethnic groups[7]. However, the interaction between ethnicity and health care use is complex and unpredictable. Recent evidence from Andean migrants in the UK shows that even within this seemingly homogeneous group very diverse health identities were constructed [8], and research on the interdependence of ethnicity and health care topics such as autonomy or EoL preferences has yielded contradictory results[9]. Therefore, when doing research or implementing EoL care services in these communities, a number of issues are of interest. Above all, cultural stereotypes have to be avoided. Minority groups themselves may develop and practice a variety of religious rites or traditions which are sources of dignity and feeling at home[10].

In Germany, several waves of immigration–late 19^th^ century Polish miners; post-World War II German-born exilees from Eastern Europe; Italian, Turkish, Spanish and Greek foreign workers recruited from 1955 to 1973 (the so-called “Gastarbeiter”); political refugees from Iran (1980s) and the former Yugoslavia (1990s); re-patriates from former Soviet territories during the 1990s; and recently Middle East civil war refugees–have formed a culturally diverse spectrum of minority communities[11]. Currently, 21% of Germany’s residents have a migration history while 11% are of foreign nationality[12, 13]. According to the recent United Nations Department of Economic and Social Affairs report[14], Germany is the top ranking immigration country in Europe, and due to a long-standing interaction of a broad range of social, political and cultural factors, has become a ‘superdiverse society’[15]. Immigration legislation (2005) and health insurance coverage of palliative care at home (2007) have raised public awareness also for health care related issues in a migrant population now coming of age. Several studies captured the needs of migrant groups that were defined by ethnicity and language. Recently, expert interviews revealed patterns of palliative care use in persons with Turkish or Arabian background in Lower Saxonia, Germany[16]. Patient-, health care-, and community-related barriers to access were identified, and policy guidelines were published. The authors emphasized that the results were “not group-specific but (…) probably valid in all patients with a migration background” and that a culture specific cookbook-approach might be misleading[17]. Instead of classifying cultures, newer concepts of cultural competency therefore focus on communication styles, social issues, and health beliefs that may be present across different cultures[18].

Therefore, the aim of this study was to explore the EoL preferences in patients with life-limiting disease with or without migration background, to elicit possible migration-related issues while avoiding cultural bias, and to generate a theoretical hypothesis for further research and clinical applications.

## Methods

The main goal of our study was to understand the needs and resources on single patient level to generate hypotheses for further research. Therefore, a grounded theory approach was chosen “to elicit each participant’s interpretation of his or her experience” [19]. Kaufmann’s grounded theory aims to move from thoughtfully conducted interviews, objective analysis, critical reduction, empirically grounded questions, and discoveries towards generating a new theory that helps to understand the social situation under examination[20]. An in-depth literature review was postponed until the data collection was completed to prevent introducing bias and perceived notions [21]. We followed the grounded theory framework proposed by Kaufmann[20] that traditionally refrains from using guidelines during the interviews, as guidelines may contradict the principle of open(minded)ness. Furthermore, it has been demonstrated that a structured interview frame may suppress natural communication and foster misinterpretation[22]. The hallmark of Kaufmann’s approach is the ‘understanding interview’ (sometimes referred to as ‘focused interview’) in which the researcher engages in a mutual conversation with the interviewees to allow them to voice their individual views, values and experiences. For this study involving migrants with life-threatening disease, this method was chosen for its capacity to facilitate trust and willingness to talk about sensitive life events.

For participation in the study, adult patients with life-threatening, far advanced and/or progressive illness receiving palliative care in different settings were included, both with and without migrant background. The sampling of negative cases (persons without migration background) was performed to avoid the bias of falsely attributing findings to ethnicity, culture, or migration background. To allow for maximum diversity of the sample, the investigators (A) enrolled people with different ethnic and linguistic backgrounds, language skills, citizenships, nationalities, and migration stories; (B) residents from urban and rural areas were recruited, and (C) institutions representing the entire palliative and hospice field (inpatient palliative care facilities, nursing homes, and home care) were approached. Patients were enrolled until data saturation was reached. The characteristics of the participants are shown in **Table 1**. Of note, all participants were actively receiving palliative care.

**Table 1:** Participants’ characteristics.

Age[years]: median (range)		70 (32–95)
Gender		25 f, 12 m
Diagnosis	Cancer	28
	chronic, non-malignant disease	9
WHO performance status: median (median)		3.5 (2–4)
Area of residence	Urban	33
	Rural	4
Living alone		17
Persons with migration background*/German natives		18/19
Migrants: residence in Germany[years]: median (range)		30 (4–52)
Place of care	inpatient hospice	5
	nursing home	2
	palliative care unit	2
	palliative home care	28
Receiving palliative care for[months]: median (range)		3 (1–36)

*includes all people having immigrated into Germany since 1949 and their offspring

Countries of origin were Belarus, Bosnia-Herzegovina, Bulgaria, Croatia, Czech Republic, Hungary, Indonesia, Israel, Lithuania, Tunisia, Turkey, Ukraine, and the U.S.; religious denominations were not routinely recorded. From February to October 2016, 48 patients were approached for participation. Of those, 4 were reluctant to think about and to discuss EoL matters, 3 declined audio-recording, 2 were too ill to do the interview, and 2 had a non-scheduled hospital appointment. Thus, 37 interviews were conducted with a mean duration of 60min each (range 40–120). Most interviews took place at the patients’ homes (see **Table 1**). In 10/37 cases, informal caregivers were present during the interview, which was taken into account as a confounding factor[23].

The study protocol was approved by the ethical review board of the Ludwig Maximilian University, Munich (No. 709–15). Screening of eligible patients was done by local palliative care staff while inclusion was done by the investigators (telephone contact). Purpose and nature of the study were thoroughly explained to the interviewees by an information leaflet and oral on-site communication. Informed consent including permission to audio-record was obtained; persons who declined audiotaping were not included in the study. Participants were encouraged to talk about their life experiences, perceptions of being ill, and perspectives regarding their current situation. To ensure the open nature of the interviews according to Kaufmann[20], the researcher intervened only minimally. Interviews were audio-taped, transcribed, and anonymized. Any additional information relevant to our study was captured in written interview protocols; reflective notes were taken on different issues that emerged when conducting the study. The complete data set (‘rich data’) thus consisted of the patient interviews, interview protocols, and reflective notes.

To present the essence of the participants’ experiences, thoughts, and feelings, the interview transcripts were organised and analysed thematically. By its nature, thematic analysis is an ongoing exploration and “a reiterative process without finite interpretation”[21]. This might appear as a very primitive way of interpreting data; however, thematic analysis is a key methodology when it comes to detecting new patterns and generating hypotheses and theories[24]. Besides identifying patterns that are communicated directly, thematic analysis allows to detect themes that are 'non-observable' in terms of language use (e.g. socially suppressed topics) and that may go undetected otherwise. [25] Such ‘non-observable’ themes may be critical in understanding a complex situation and refine the theoretical concepts under examination.

For this study one researcher (PP), who conducted all interviews and was actively involved in transcribing the recorded data, constantly moved back and forth between conducting the interviews and analysing the data (in-vivo coding and categorization) until no new patterns emerged. The simultaneous data collection and data analysis process allowed asking questions that helped to elicit the significance of a specific issue in subsequent interviews (e.g. applying migration-specific themes in non-migrant participants). During the coding process the understanding of findings and their relevance changed, expanded, and deepened, which resulted in creating the theoretical categories. To ensure the consistency of findings, a second investigator (JB) double-checked the transcripts and in-vivo codings before proposing the final categories. Qualitative data analysis software MaxQDA 12 was used.

## Results

**Fig 1:**
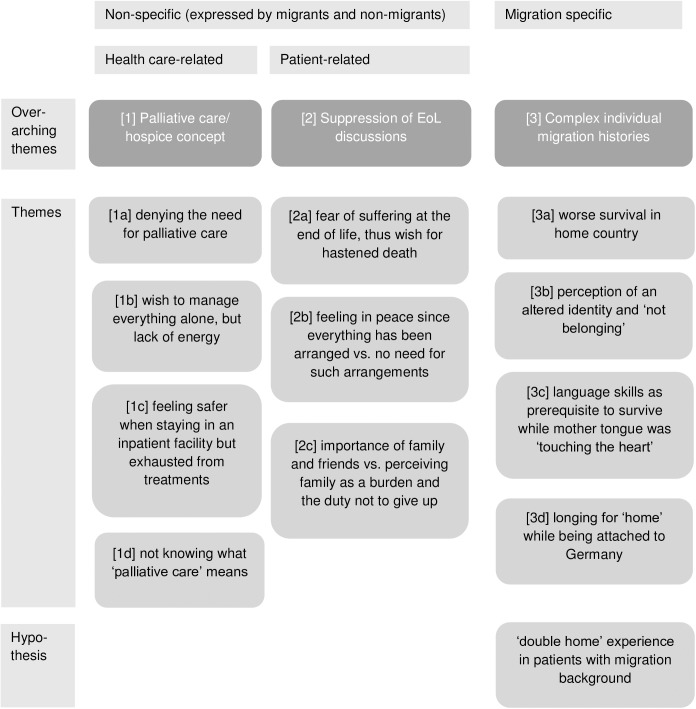
Final themes grouped according to migration background and predominant relation to health care / patients’ issues.

An overview of all themes grouped according to migration background and health care/patient related issues is provided in **Fig 1**, and examples are shown in detail below. For reasons of limited space, the main focus of this paper is on the migration-specific themes.

Three overarching themes (1, 2, 3) were repeatedly expressed by both migrant and non-migrant patients. From the subsequent themes, 1a-2c were expressed by study participants from both groups while 3a-d were specific for participants with a migration background. Themes 1a-d are predominantly health-care related while 2a-c consist of more personal and psycho-social issues; however, there is considerable overlap.

(1) The understanding of palliative care was generally limited, and concepts that significantly deviate from the official WHO definition became apparent. For example, the term ‘palliative’ was associated with terminal care and assisted suicide for some patients. Moreover, high expectations regarding curative treatment indicated that the majority of patients was not familiar with the underlying concept of palliative care, they had never heard the word ‘palliative’, and had no knowledge of the available palliative care services. ‘Hospice’ was familiar to more participants and associated with terminal care and ‘peaceful dying surrounded by friends and family’.

(1a) Some patients clearly felt that their condition would at that time not require the involvement of palliative care services. This was mostly due to curative treatment preferences or misconceptions of what palliative and hospice care were offering.

‚First, I thought: ‚My god, I am going to hospice care–I am really not yet on the road to death!’ (Woman, *1969)

(1b) Participants wished to live their own lives and to manage everything on their own as they had been used to so far. At times they refused support by health care services (home care nursing, mobile palliative care teams) despite progressive fatigue, weakness, and lack of endurance.

‚Sometimes I am feeling totally unsteady but somehow I’ve always managed it.‘ (Woman, *1957)

(1c) For that reason, some interviewees argued that staying in a hospital would give them a feeling of safety, and some felt obliged to consult various specialists for effective symptom control or prolongation of life. At the same time, however, they admitted to be tired of therapeutic interventions which were aggressive, burdensome, and repetitive. This ambiguity repeatedly was triggering conflicts in patients and their families.

‚Don’t want to go on with these devices… there are no chances, I know, for me. I’m waiting.’ (Man, *1945)

(1d) To some participants, the meaning of ‘palliative care’ remains elusive or they had not heard of that term so far. This lack of knowledge led to misconceptions and under-use of palliative and hospice care.

(2) The concept of EoL seemed to be rejected by the majority of participants as it was associated with fear, loss of autonomy, and suffering. They felt uncomfortable or even offended whenever ‘EoL’ was mentioned. Thus, EoL issues were reluctantly addressed, and preferences regarding EoL decision making were expressed only with great cautiousness. Frequently, interviewees denied that their own EoL might be approaching, they rather felt ‘they were not that far yet’. While ‘EoL’ was perceived as a very frightening period of time in the future and thus avoided, patients discussed ‘death’ and ‘dying’ more openly, as for them, those terms implied an endpoint, the termination of suffering, and salvation. Thus, ‘EoL’ and ‘death/dying’ are not used synonymously.

(2a) Talking about EoL was problematic due to the fear of suffering, but people openly discussed their wish for hastened death and hoped that when they reached that point, a palliative care team or people in hospice would take care of their final wish:

‚I hope that I will be getting a drug so I’m asleep today and the day after tomorrow this will be over. And I hope that that institution will help me with, let’s say medications.’ (Woman, *1948)

‚When you are sick, you cannot concentrate, that’s impossible. Then you have always got the feeling (…) that you rather want to die.‘ (Woman, *1961)

It became evident that patients were most afraid of what would happen to them in the EoL phase before the final salvation. Therefore, EoL discussions and decision making were regarded as difficult. Frequently, concerns and worries were expressed only between the lines (e.g. reluctance to talk about the EoL), and talking about receiving palliative or hospice care or being treated in/by such an organisation is linguistically and socio-culturally suppressed.

(2b) Some participants reported they ‘felt in peace since everything had been arranged’ while others denied a need for making such arrangements. Individualized advance care planning, arranging the funeral, deciding on financial matters, and addressing other social, existential or spiritual issues seemed to be a source of comfort to these patients.

(2c) The presence of family members and friends was generally perceived as a very potent resource of comfort and support. It stimulated a will to survive and ‘a duty not to give up’. However, also the opposite was recorded in some cases, i.e. relatives being an additional burden to the patient.

‚That hurts terribly: I had wanted to see my grandson grow up, he’s only 10…’ (Woman, *1954)

‚I have to get stabilized somehow, I have to go on living, because I have a son and he needs me. He is 15.’ (Woman, *1969)

(3) The distinction line between participants with or without a migration background was rather blurred than clear-cut. When asked for their own migration background, they revealed complex personal migration histories–e.g. being a German resident for decades but admitting later during the interview to be born and raised abroad; having changed citizenship not due to migration but to post-World War II reorganization of national borders; or being a member of a native German minority that had remigrated from former Soviet territories. In some participants, the migration background was almost undetectable. When the typical signs, such as poor language skills, different looks, exotic names etc. are missing, migration history is easily overseen. On the other hand, a patient might not have any individual migration history albeit having all signs of migration background. In this study, some patients with migration background called Germany their home country, while some felt stuck between their German home and their home elsewhere.

(3a) Patients who had immigrated from a region with limited health care availability (according to their individual perception) knew very clearly that they might not be alive any more in their country of origin. They were aware that they needed to stay in Germany to receive appropriate care which they gratefully appreciated.

‚I have a friend who is a doctor, and he told me, If I had stayed in Russia than I would have been ruined and not alive any more. Because the medical system is wrecked. I am thankful to be in Germany with my illness. These 8, 9 years have been a good time, made possible by medicine and good care.’ (Woman, *1946)

(3b) A wide range of expressed views refers to an altered identity. The migration history had clearly altered the patients’ self-perception. Some of them felt fully integrated and even complained about their fellow countrypersons’ manners:

‚How can you speak Bosnian that loudly in the subway–it’s impossible, it’s annoying! (…) You have to adapt respectfully to the culture and everything is ok, that’s what I think!’ (woman, *1962)

However, patients also experienced difficulties and barriers regarding culture, habits, and language. Thus, they were not sure where they essentially belonged to or reported a general feeling of ‘not belonging’:

‚And when I’m in Turkey–well, (…) you are not alone. If you get out of your door, your neighbors are there already. One of them is making tea, the other one börek, you are sitting together and sharing everything–that’s wonderful! But here in Munich? I don’t even know my neighbor. (…) I wish my father had never come here.’ (Woman, *1963)

(3c) Language skills were emphasized to be a prerequisite to survive in a foreign health care system. A major challenge to patients with migration background was to process vital information about prognosis and therapy in a foreign language (German) while suffering from distressing symptoms. On a more emotional level, they expressed that the mother tongue was ‘touching the heart’.

‚When I’m not well, for example, I have to struggle with another language which means more stress. Sometimes I cannot think at all. I start to talk and am forgetting the words, which is from the therapies. My son is asking: don’t you know any more how to speak German? I say, I don’t know, it’s all mash in my head and when I have pain I’m going and thinking into another language…’ (Woman, *1969)

(3d) The underlying theme was a ‘double home’ experience. Although in Germany the patients’ families and other social contacts were present, patients were at times still missing their home in the place of their origin:

‚No. I want to stay with my wife, my family… Our home country is Romania, where my parents lived, no problem to visit when I’m fine. (weeping) But no Romanians are going back. My family is my wife and my children, I want to be together with all of my family. Impossible… I tell you: my country has never helped me as Germany did. That’s the way it is.’ (Man, *1948)

The interviewees were reflecting on homesickness and what the host country–which the majority of them called ‘home’ as well–was meaning to them:

‚Home sickness? Don’t have that. I am here at home, sort of… because since 1968, that’s been more years than in Czechoslovakia. My daughter and friends are here–more people than there, and four of my people have died here.’ (woman, *1937)

‚Somehow, I have a positive feeling towards cremation. And if: perhaps part of the ashes here and part in Bulgaria, because I feel at home there and here also by now. Two homes: there and here.’ (Woman, *1969)

## Discussion

This study demonstrates that people with incurable illness and migration background present with migration specific issues at the EoL in terms of health care use, language, and identity. However, these data from a country with substantial immigration show that the socio-political construct of ‘migration background’ alone is not helpful to predict such issues and needs in individuals. This construct may obscure alternative explanations for a problem (e.g. social deprivation), thus leading to false assumptions. The findings include three overarching themes: (1) limited understanding and knowledge of palliative care and hospice across the entire group; (2) a suppression of the concept of ‘end of life’, also seen in both groups; and (3) complex individual migration histories that may blur the distinction between being a ‘migrant’ or ‘non-migrant’. Given this fuzziness, seven non-specific themes emerged which covered health care (1a-d) and patient related issues (2a-c), while four themes were specific for patients with migration background (3a-d). Migrants emphasized that both access to health care and survival were generally worse in their country of origin (3a); they expressed issues of altered identity (3b) and language use (3c) on practical and emotional levels; and they provided narratives of their ‘double home’ experience (3d).

The data presented here fit into the international context. According to a recent focus group study in Sweden, health care professionals were regarding persons with a migration background as ‘unusual’ or ‘the unknown’, and they were talking about them in a homogenizing manner[26]. Moreover, they felt unable to deliver culture-sensible care and regarded migrant patients as ‘others’[27]. In contrast, by directly approaching the patients, this study revealed that in some cases the individual ‘migration background’ was difficult to assess or even invisible (overarching theme 3). Thus, if ‘otherness’ was the typical feature of a migrant, migrant specific needs might remain unknown and might be not addressed by health care professionals. By its holistic and person-centred nature, the palliative care approach may be particularly sensitive to the needs of a culturally and socially diverse population[28]. However, this study is revealing barriers and limitations in the understanding and use of palliative care (overarching themes 1 + 2). These findings are supported by a number of studies–one example being Turkish and Moroccan immigrants in the Netherlands who thought the concept of ‘good palliative care’ to be contradictory: for them, ‘good care’ was synonymous with intensive and curative treatment[29]. But such concepts are ‘fluid and mutable’, persons may adopt multiple cultural and social identities according to changing circumstances, and wide heterogeneity has been observed within any given cultural group[8, 30].

Limitations of this study are first of all due to sampling: interviewees had been recruited within palliative care settings, which may have excluded ‘palliative-naïve’ voices. In addition, most participants were living in Germany for decades and speaking German fluently–recently arrived refugees, for example, may have expressed different perspectives. Secondly, a study using qualitative methodology is generating hypotheses which require further testing, i.e. causal conclusions may not be drawn from the findings. Furthermore, the results have to be compared with those from other countries although the data have been collected in an urban area of a country with a large, representative immigrant population.

However, the paramount strength of this work is the presentation of the patients’ own perspectives. Most studies so far have approached surrogate respondents such as health care experts or relatives[6, 17, 26], as patients seemed to be too vulnerable and sick to participate. Secondly, the sample size was large enough to represent the diversity seen in an urban population. A third unique point is the novel two-armed study design enrolling both migrants and non-migrants. This allowed to identify migrant-specific themes and to avoid ethnic or cultural stereotyping of phenomena. And fourth, the open interviewing technique and thematic analysis enabled the researchers to detect hidden themes such as misconceptions of ‘palliative care’ [7] and the difficulty to talk about the EoL [8].

### The ‘double home’ experience

Further research has to start from the themes expressed by the patients in this study. Being stuck between their German home (e.g. for medical reasons) and their home elsewhere, the meaning of ‘being at home’ and ‘dying at home’ may cause distress, ‘social pain’[31], and homesickness. Physical, family, financial, or legal restraints may keep patients from returning to their home country. A simple question such as “Besides this country, is there another place where you feel at home?” may sensitively screen for these complex, migration-related issues, which require an adequate response by health care. The authors propose a theory of ‘double home’ experience, which is interconnecting the themes expressed by patients with migration background (see **Fig 1**). Therefore, next steps will be to test this hypothesis in a quantitative survey using items derived directly from the themes of this study, and to prospectively evaluate the screening question.

### The risk of ‘othering’

While terminally ill patients with a ‘double home’ experience have similar views, concerns and preferences regarding care at their EoL as patients without this experience, they may easily become subject to ‘othering’[26]. In a diverse population, culture-based presumptions need to be challenged in order to deliver high quality care to all patients regardless of their background. It is therefore essential to understand the patient’s individual experience and address aspects of ‘social pain’[31]. The American Academy of Family Physicians has issued clinical guidelines that also acknowledge communication patterns deviating from common practice, e.g. regarding disclosure of prognosis and decision making[32]. For health care professionals, core recommendations therefore include: paying attention to language and communication, improving the knowledge of palliative care services in the communities, reflecting on what ‘cultural competency’ is meaning, and being aware that cultural stereotypes may impair the quality of care[2].

## Supporting information

S1 Table Overview of codes on single patient level. Left column: codes (German). Each dot is representing a code being expressed by one patient, the size of dots indicating the number of times the patient was using this code. Diagram by MaxQDA 12 software.
(XLSX)

S2 Table From codes to themes and overarching themes. German codes were left in their original style, including grammatical errors (1^st^ column). English versions and additional comments are provided in the 2^nd^ and 3^rd^ column, themes and overarching themes are corresponding to Fig 1.
(XLSX)
